# Landscape exposure and exercising for health: exposure to natural versus urban landscapes promotes walking for health

**DOI:** 10.1080/00049530.2025.2450351

**Published:** 2025-01-19

**Authors:** Hui-Ju Wu, Yevvon Yi-Chi Chang, Wen-Bin Chiou

**Affiliations:** a Center for Teacher Education, Cheng Shiu University, Kaohsiung, Taiwan; b International Business Administration Program, Tunghai University, Taichung, Taiwan; c Institute of Education, National Sun Yat-sen University, Kaohsiung, Taiwan

**Keywords:** Exercising for health, exposure to nature, health behaviour, temporal discounting, urban landscape

## Abstract

**Objective:**

A common tendency among humans is the devaluation of remote, larger benefits in favour of immediate, smaller gains, a phenomenon known as temporal discounting. Exercising for health necessitates focusing on long-term health benefits while minimising perceived obstacles. Recent studies have demonstrated that experiencing nature may reduce the discounting tendency. We conducted a behavioural experiment to investigate whether exposure to natural environments could decrease temporal discounting, thereby enhancing the inclination to walk for health.

**Method:**

In total, 140 participants were randomly assigned to view images of either natural or urban landscapes. They completed a measure of discounting and participated in a pedometer-based task. In this task, the selection of a meeting point at a greater or lesser distance, along with the additional distance walked, were used as indicators of health-oriented walking behaviour.

**Results:**

Participants exposed to natural settings were more likely to choose a distant meeting point for returning the pedometer and engaged in greater additional walking than those exposed to urban settings. Temporal discounting played a mediating role in these effects.

**Conclusions:**

These findings offer an explanation for the reduced propensity towards health-related exercise among urban residents, and provide a novel strategy for promoting exercise motivation in contemporary lifestyles.

## Key Points

What is already known about this topic:

(1) Recent findings suggest that exposure to nature reduces individuals’ tendency to discount the future, resulting in lower discounting.

(2) Lower discounting, a crucial manifestation of self-control, is closely related to motivation and engagement in exercise.

(3) No prior experimental studies have examined the effect of nature exposure on exercise to improve health.

What this topic adds:

(1) Experimental exposure to natural scenes increases the motivation to exercise for health benefits.

(2) The tendency to discount long-term benefits is associated with engagement in walking for health.

(3) Reduced discounting tendency, prompted by exposure to nature, underlies the relationship between nature exposure and the tendency to walk for health.

## Introduction

Numerous studies have documented the health advantages of exercise, including improvements in cardiorespiratory fitness, longevity, and mental health (Ruegsegger & Booth, 2018). Research on motivators and barriers influencing exercise participation has explored demographic and biological aspects (e.g., age, sex, body mass index, childlessness, heredity, socioeconomic factors, and race or ethnicity), physical factors (e.g., appearance, fitness, physical health, physical illness, tiredness/fatigue, strength, and weight loss), psychological factors (e.g., affective states and mood, anticipated benefits, body image, better sleep, distress, exercise enjoyment, exercise intention, health perceptions, self-control, self-confidence, self-efficacy, self-esteem, stress reduction, time management, and well-being), behavioural attributes and skills (e.g., activity history, dietary habits, engagement in past exercise programs, skills for overcoming barriers, and smoking habits), social and cultural influences (e.g., physician advice, social isolation, and social support), environmental factors (e.g., actual and perceived access to facilities, appealing scenery, neighbourhood safety, satisfaction with facilities, and location), and characteristics of physical activity itself (e.g., intensity and perceived effort; for related reviews, see Firth et al., 2016; Trost et al., 2002). Extensive evidence indicates the health impact of exposure to nature, including beneficial effects on affective state, blood pressure, brain activity, cardiovascular disease, cognitive function, immune function, mental health, mortality, physical activity, sleep, stress, and well-being (e.g., James et al., 2015; Mitchell & Popham, 2008; Thompson et al., 2012; Twohig-Bennett & Jones, 2018; Wolch et al., 2014; Wood & Smyth, 2020; also see; Jimenez et al., 2021, for a related review). Additionally, previous studies on exercising in nature, also termed “green exercise” (Shanahan et al., 2016), have primarily examined its impacts on physical and mental health (e.g., Barton & Pretty, 2010; Lahart et al., 2019; Lee & Maheswaran, 2011; Pretty et al., 2005; Rogerson & Barton, 2015; Wood & Smyth, 2020). Attention-restoration theory (S. Kaplan, 1995; R. Kaplan & Kaplan, 1989) has been extensively applied in environmental psychology to account for the beneficial outcomes of nature-based exercise (e.g., Jiang et al., 2021; Snell et al., 2019; White, Alcock, et al., 2013; Williams et al., 2018; also reviewed in; Lahart et al., 2019). Current knowledge is limited by a lack of experimental evidence on the impact of exposure to nature on exercise to improve health, an absence of behavioural measures of exercising for health, and insufficient explanation for the link between nature exposure and the inclination to engage in exercising for health. Hence, the present experimental study examined the effect of nature exposure on the tendency towards exercising for health and explored the mediating role of temporal discounting as an underlying mechanism in this relationship.

Temporal discounting indicates the tendency to prefer immediate smaller rewards over delayed larger rewards in inter-temporal decision-making (Frederick et al., 2002; Green & Myerson, 2004). Exercising for health, representing an inter-temporal choice, encompasses the pursuit of long-term health benefits (approach motivation) and the challenge of overcoming exercise barriers (avoidance motivation). The predisposition towards temporal discounting, favouring near-term over long-term motivations, has been identified as an indicator of poor self-control (Ainslie, 1975; Chiou et al., 2017; Fujita & Han, 2009; Mischel et al., 1989; see also Fujita, 2011, for a related review). Lower levels of self-control in the context of exercise implies foregoing future health benefits for immediate non-exercise motivations. Conversely, higher self-control, characterised by reduced discounting (Fujita, 2011), entails resisting the temptation to avoid exercise in favour of long-term health outcomes. Therefore, reduced discounting, which is indicative of a stronger focus on future benefits, is expected to increase commitment to health-oriented exercise.

According to the concept of environmental contingency (H. S. Kaplan & Gangestad, 2005), the natural environment can influence our decisions, feelings, and behaviours (e.g., Atchley et al., 2012; Purcell et al., 2001; Van den Berg et al., 2003). For example, experiencing the natural environment can restore attention (e.g., Berman et al., 2008; Berto, 2005), enhance mood and happiness (e.g., Bowler et al., 2010; White, Pahl, et al., 2013), reduce stress (e.g., Thompson et al., 2012; Ulrich et al., 1991), and encourage cooperative and pro-environmental behaviour (Zelenski et al., 2015). Recent studies on the link between nature exposure and temporal discounting have suggested that exposure to (or immersion in) nature may reduce the discounting tendency (e.g., Berry et al., 2014, 2015; Y. Y. Chang & Chiou, 2024; Y.-Y. Chang et al., 2024; Kao et al., 2019; Taylor et al., 2002; van der Wal et al., 2013; Wu & Chiou, 2019). Life history theory posits that individuals may respond differently to environmental signals that indicate the occurrence of opportunities and threats (H. S. Kaplan & Gangestad, 2005). Specifically, individuals are less likely to discount the future in environments perceived as predictable or non-competitive, whereas they are more likely to discount the future in environments seen as unpredictable or competitive (Ellis et al., 2009; Griskevicius, Tybur, et al., 2011; R. Kaplan & Kaplan, 1989). Natural landscapes, particularly lush ones, may signal predictability and resource abundance (Griskevicius et al., 2012), leading individuals to discount the future to a lesser extent (Berry et al., 2014; van der Wal et al., 2013). Conversely, urban landscapes may signal unpredictability and social competition for resources (e.g., status, goods, and mates), leading to steeper discounting of the future (Ellis et al., 2009; Griskevicius et al., 2012; van der Wal et al., 2013).

### The current research

In summary, based on the influence of natural vs. urban landscapes on temporal discounting and its relation to the propensity for health-oriented exercise, we hypothesised that exposure to nature would reduce the discounting tendency, leading to an increased inclination towards exercising for health. A behavioural experiment was conducted to test the connection between viewing pictures of natural (vs. urban) landscapes and a higher tendency of engagement in walking for health, and to examine whether temporal discounting would serve as the mediating role in this connection.

## Method

### Participants

One hundred forty college students (72 females, 68 males) participated in this study. The sample size was based on a calculation to ensure adequate power for conducting an independent-samples Student’s *t*-test across two study conditions, with the set parameters α = 0.05, effect size (Cohen’s *d*) = 0.50, and power (1 – β) = 0.90 (Kirk, 2012). Recruitment was facilitated through online and campus advertisements. The current study was approved by the Research Ethical Committee of Kaohsiung Medical University in Taiwan.

### Procedure and materials

Upon their arrival, participants were briefed and provided their consent. Initially, we measured each participant’s body mass index (BMI) and evaluated their motivation to exercise for health using a 7-point scale, from 1 (*not at all*) to 7 (*very high*). Subsequently, pairs of same-sex participants were randomly assigned to one of two conditions: natural landscape or urban landscape. We maintained identical sex ratios across conditions to help control for potential sex effects. Landscape exposure consisted of viewing five pictures of either natural or urban scenes on a laptop screen, with each image displayed for 2 min. These images had been validated for their respective environmental contexts in a pilot study and previous research (Kao et al., 2019; Wu & Chiou, 2019). Participants were instructed to immerse themselves in the depicted landscapes. For the manipulation check, participants rated the naturalness and urbanness of the landscape pictures on a 7-point scale ranging from 1 (*not at all*) to 7 (*very much*), with the order of questions counterbalanced. They also assessed their level of immersion in the landscapes on a similar 7-point scale.

Subsequently, participants completed a discounting measure. In this task, they made choices between immediate and delayed monetary rewards by responding to nine binary questions (e.g., “A. Win NT $2,000 immediately; B. Win NT $2,283 one year from now”). Choices varied between receiving NT $2,000 now or different amounts ranging from $1,883 to $4,000 in one year. The future amounts were presented in a fixed ascending order (Rodzon et al., 2011). Following the approach of Hardisty and Weber (2009), the discounting rate was calculated for each participant using a hyperbolic discounting formula: *k* = (*A/V* −1)/time in 1 year, where *A* represents the smallest future amount deemed equivalent to the present value *V*. For example, a future amount of $3,717 yields a discounting rate of .8585 (i.e., 3,717/2,000 − 1). Lower *k* values indicate a stronger preference for delayed over immediate delayed rewards, reflecting lower discounting tendencies.

Regarding the task of walking for health, participants were instructed to assist in testing a pedometer under the guise of another purpose. This task entailed walking to one of two well-known landmarks on the university campus and returning the pedometer to an experimenter. Subsequently, participants were required to read a medical report highlighting the relationship between walking and health benefits. This procedure was designed to prime the participants’ motivation for healthful exercise and assess the durability of the nature exposure effect. Participants were informed that they had 40 min to deliver the pedometer to either of the designated meeting points. During this time, they were free to walk anywhere or visit anyone en route to the meeting location. The primary measures of interest were the chosen meeting point (~500 or ~ 1,000 m away) and any additional distance covered. The additional distance walked by the subject was determined by subtracting the physical distance to the selected meeting point from the distance recorded by the pedometer. At the conclusion of the experiment, participants were allowed to inquire about the research objectives. None accurately identified the true purpose or the interconnection between the tasks. Debriefing was conducted via email five days after the experiment concluded to prevent participants from revealing the actual intent to their peers.

## Results

### Manipulation check

To determine whether nature landscape pictures were perceived as more or less natural than urban landscape pictures, paired-sample *t*-tests were conducted. Nature landscape pictures were rated significantly more natural (*M*_nature_ = 6.13, *SD* = 0.58) than urban landscape pictures (*M*_urban_ = 1.85, *SD* = 0.56; *t*(138) = 44.618, *p* < 0.001, Cohen’s *d* = 7.51, 95% confidence interval (CI) of the mean difference: 4.09 − 4.47) and significantly less urban (*M*_nature_ = 1.82, *SD* = 0.56) than urban landscape pictures (*M*_urban_ = 6.14, *SD* = 0.56; *t*(138) = −45.692, *p* < 0.001, *d* = 7.71, 95% CI of the mean difference: −4.51 to − 4.13). Participants in the natural condition reported significantly higher landscape immersion (*M*_nature_ = 5.69, *SD* = 0.83) than the midpoint of the immersion scale (4.0; *t*(69) = 17.073, *p* < 0.001, *d* = 2.04, 95% CI of the mean difference: 1.49 − 1.88), as did participants in the urban condition (*M*_urban_ = 5.73, *SD* = 0.72; *t*(69) = 20.066, *p* < 0.001, *d* = 2.40, 95% CI of the mean difference: 1.56 − 1.90). These results confirm the efficacy of landscape manipulation. Additionally, there were no significant differences in participant BMI (*M*_nature_ = 22.63, *SD* = 3.66; *M*_urban_ = 23.18, *SD* = 4.23; *t*(138) = −0.820, *p* = 0.413) or motivation to exercise for health (*M*_nature_ = 3.81, *SD* = 1.56; *M*_urban_ = 3.96, *SD* = 1.62; *t*(138) = −0.531, *p* = 0.596) between the two study conditions (Table 1), suggesting that the random assignment process effectively created equivalent groups. Therefore, these variables were not controlled for in further analysis.

**Table 1.** Descriptive statistics for each measure.

	Natural scene		Urban scene	
Measures	Mean	95% CI	Mean	95% CI
BMI (kg/m^2^)	22.63	[21.76, 23.50]	23.18	[22.17, 24.19]
Motivation to exercise for health (1–7)	3.81	[3.44, 4.18]	3.96	[3.58, 4.34]
Discounting rate (*k* parameter)	0.45	[0.39, 0.51]	0.62	[0.57, 0.67]
Additional distance walked (m)	165.63	[152.24, 179.02]	127.19	[114.18, 140.20]
Choice of the farther meeting point (%)	53	[41, 65]	26	[16, 36]

Each condition involved 70 participants. Units of the dependent measures are presented in parentheses. BMI = body mass index; CI = confidence interval.

### Temporal discounting

As shown in Table 1, participants exposed to the natural landscape exhibited a lower mean discounting rate (*M*_nature_ = 0.45, *SD* = 0.24) compared to those in the urban landscape condition (*M*_urban_ = 0.62, *SD* = 0.21; *t*(138) = −4.441, *p* < 0.001, *d* = 0.75, 95% CI of the mean difference: −0.25 to − 0.09). This outcome suggests that nature exposure leads to a decreased tendency for temporal discounting. Furthermore, the effect of landscape exposure on discounting rates was not influenced by participant sex (*F*(1, 136) = 0.653, *p* = 0.420), indicating that the nature-induced reduction in discounting was consistent across both female and male participants.

### Walking for health

In relation to the selection of the meeting point (0 = nearer; 1 = farther), Table 1 shows that choosing the farther meeting point was significantly associated with the experimental condition (0 = urban; 1 = natural; Table 1, χ^2^ (1, *N* = 140) = 10.811, *p* = 0.001, *φ* = 0.278). Logistic regression analysis revealed that participants in the natural landscape group were more likely (53%, 37 of 70) to choose the farther meeting point compared to participants exposed to the urban landscape (26%, 18 of 70; *B* = 1.18, standard error (*SE*) = 0.36, Wald = 10.455, *Z* = 3.233, *p* = 0.001, Negelkerke *R*^2^ = 0.102; odds ratio (OR) = 3.24, 95% CI: 1.59 − 6.60). Furthermore, participants in the natural landscape condition engaged in significantly more additional walking en route to returning the pedometer (*M*_nature_ = 165.63 m, *SD* = 56.17) than those in the urban landscape condition (*M*_urban_ = 127.19 m, *SD* = 54.56, *t*(138) = 4.107, *p* < 0.001, *d* = 0.69, 95% CI of the mean difference: 19.94 − 56.95; Table 1). The interaction of condition by sex regarding the additional distance walked in the pedometer task was not significant (*F*(1, 136) = 0.166, *p* = 0.684), indicating that the influence of nature vs. urban exposure on walking for health was consistent across both females and males.

### The mediating role of temporal discounting

The four-step process for testing mediation, as outlined by Baron and Kenny (1986), was used to assess the mediating role of discounting rates on the two dependent measures (i.e., the selection of the more distant meeting point and additional walking). The analysis for the choice of the meeting point revealed that exposure to nature significantly predicted the discounting rate (*B* = −0.17, *SE* = 0.04, *t* = −4.441, *p* < .001), the discounting rate significantly predicted the choice of the farther meeting point (*B* = −5.18, *SE* = 1.01, Wald = 26.179, *Z* = 5.117, *p* < 0.001), and the effect of exposure to natural scenes on the choice of the farther meeting point became non-significant (before control: *B* = 1.18, *SE* = 0.36, Wald = 10.455, *Z* = 3.233, *p* = 0.001; after control: *B* = 0.56, *SE* = 0.42, Wald = 1.831, *Z* = 1.353, *p* = 0.176) when including the discounting rate in the model. Bootstrap analysis (Preacher & Hayes, 2004) also indicated a significant indirect effect, with a 95% bias-corrected CI (0.37–1.42) for the indirect effect (*B* = 0.85, *SE* = 0.28; 5,000 bootstrap samples) not including zero, suggesting a significant mediation by the discounting tendency on the connection between exposure to natural scenes and choosing the more distant meeting point.

**Figure 1.**
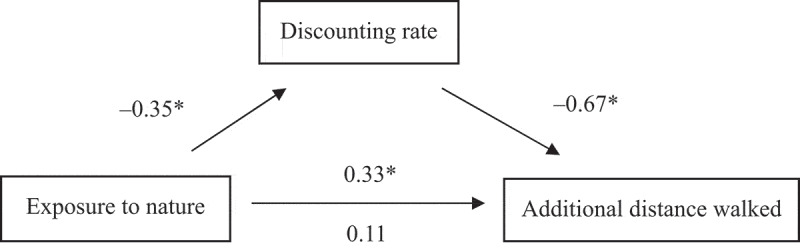
The discounting tendency mediated the association between nature exposure (1 = viewing pictures of natural landscapes, 0 = viewing pictures of urban landscapes) and additional distance walked (m) in the pedometer task. Path values are standardized regression coefficients. On the lower path, the values below and above the arrow are the results of analyses in which the mediator was and was not included in the model, respectively. An asterisk indicates a *p*-value of less than 0.05.

Regarding additional distance walked, exposure to natural scenes was associated with lower discounting (*B* = −0.17, *SE* = 0.04, *t* = −4.441, *p* < 0.001), and lower discounting was linked to more additional walking (*B* = −161.79, *SE* = 15.26, *t* = −10.603, *p* < 0.001). The connection between nature exposure and additional walking became non-significant (before control: *B* = 38.44, *SE* = 9.36, *t* = 4.107, *p* < 0.001; after control: *B* = 12.39, *SE* = 7.83, *t* = 1.584, *p* = 0.116) when the discounting rate was controlled for (Figure 1). Bootstrap analysis confirmed a significant indirect effect (*B* = 26.03, *SE* = 6.10, 95% bias-corrected CI: 14.65–39.13; 5,000 bootstrap samples).

## Discussion

Expanding on the established link between nature exposure and a lower rate of discounting (Berry et al., 2014, 2015; Y. Y. Chang & Chiou, 2024; Taylor et al., 2002; van der Wal et al., 2013), as well as the relation of lower discounting to increased engagement in willpower-dependent behaviours (Appelhans et al., 2012; Chiou et al., 2013, 2015, 2017; Daniel et al., 2013; Kuo et al., 2016; Reynolds, 2006), we conducted a behavioural experiment to examine the hypothesis that exposure to natural (vs. urban) landscapes would correlate with lower discounting, potentially leading to a higher inclination towards engaging in health-promoting walking activities. Our results supported these hypotheses, demonstrating that participants exposed to images of natural landscapes exhibited lower discounting rates and engaged in more walking in a pedometer task than those exposed to urban landscapes. To our knowledge, this study is the first to illustrate a link between experimental exposure to nature and the adoption of health-promoting behaviours.

The observed link between exposure to nature and reduced discounting aligns with previous research indicating that interaction with natural environments fosters delayed gratification and a higher valuation of long-term benefits (Berry et al., 2014; Y.-Y. Chang et al., 2024; Taylor et al., 2002; van der Wal et al., 2013). The inclination towards using slower strategies upon exposure to nature can be drawn from the concept of environmental contingencies (Berry et al., 2015; Griskevicius, Delton, et al., 2011; Griskevicius, Tybur, et al., 2011). Natural landscapes, characterised by cues that suggest resource abundance and predictability (Berry et al., 2014; van der Wal et al., 2013), may promote a far-sighted strategy, leading individuals to discount future benefits to a lesser extent. Conversely, urban landscapes, which are marked by cues indicative of scarce and competitive resources (Berry et al., 2014, 2015; van der Wal et al., 2013), might prompt a short-sighted approach, causing a greater tendency to discount future benefits more heavily (Ellis et al., 2009). These findings enrich the body of literature on the connection between exposure to nature and discounting tendencies.

Exposure to natural (vs. urban) scenes was linked with the selection of a farther meeting point in the pedometer-returning task, reflecting engagement in health-promoting walking behaviour. The ability to control indulgence and overcome laziness is crucial for participating in exercise (e.g., Englert & Rummel, 2016; Lindwall & Martin Ginis, 2006; Schöndube et al., 2017; Yu et al., 2022). Our results suggest that reducing discounting rates, which is indicative of greater self-control as triggered by viewing natural landscapes, fosters a greater inclination towards health-promoting behaviours. This study illuminates an effective strategy for encouraging exercise engagement, showcasing the beneficial use of nature exposure for health-oriented physical activity. We determined that the discounting rate mediates the impact of nature exposure on walking for health, as demonstrated by the selection of a farther meeting point and increased activity in the pedometer task. Considering that engaging in exercise requires overcoming inertia or barriers to achieve health benefits, our mediation analysis indicates that exposure to natural (vs. urban) scenes can facilitate walking for health by enhancing focus on the long-terms benefits of exercise. Additionally, the mediating role of temporal discounting in the association between exposure to urban scenes and a diminished tendency towards walking for health explains the lower likelihood of exercise among individuals in urban settings (e.g., Caspersen et al., 2000; Humpel et al., 2002; King et al., 2000; Ross, 2000; Wilcox et al., 2000).

The current research demonstrated that the positive effect of exposure to natural scenes on the inclination to walk for health was mediated by lower discounting. Considering that a lower tendency towards temporal discounting reflects a future-oriented mindset (e.g., Cheng et al., 2012; Macaskill et al., 2019; Scholten et al., 2019; Shevorykin et al., 2019), interventions designed to stimulate such a mindset can reduce the discounting tendency. For example, Cheng et al. (2012) found that priming individuals with future prospects increased their preference for long-term benefits over immediate gains (i.e., a lower tendency towards temporal discounting). Kuo et al. (2016) demonstrated that interacting with a computer-generated image of one’s weight-reduced self (i.e., an avatar of the ideal self) was associated with lower discounting rates. Furthermore, engagement in episodic future thinking, which involves mentally projecting oneself to pre-experience a future event (Atance & O’Neill, 2001), has been shown to reduce the discounting tendency (e.g., Chiou & Wu, 2017; Peters & Büchel, 2010; Wu et al., 2017). In the context of nature exposure, the aforementioned studies suggest that a hybrid intervention combining exposure to vivid natural environments (e.g., through the use of virtual reality or augmented reality technologies, landscape videos, or real environments; for a review, see Browning et al., 2021) and episodic future thinking that emphasises a future healthy self could amplify the beneficial effects of nature exposure (or immersion) on exercise for improved health.

This study had several limitations. Our dependent measures, including the choice of a farther meeting point for pedometer return and additional walking, may have limited the generalisability of our findings to broader health-related exercise contexts. Future studies should incorporate actual physical activity, such as exercise training sessions or daily exercise logs, to enhance applicability. The participant pool consisted solely of college students, suggesting the need for research involving a broader demographic. The present study employed a monetary choice task to assess the discounting tendency. Future research should explore alternative measures of discounting, such as the area under the receiver operating characteristic curve (Myerson et al., 2001) and scenarios related to non-monetary outcomes such as environmental or health gains (e.g., Hardisty & Weber, 2009; Joshi & Fast, 2013), to provide convergent validation. Although our random assignment procedure appeared to establish equivalent groups, the absence of measurements for potential factors (e.g., health state, affective states, trait self-control, and the initial rate of temporal discounting) that could influence engagement in exercise is a limitation that must be acknowledged. These potential mediators or moderators of the relationship between visual exposure to natural landscapes and exercise for health warrant further investigation. Additionally, similar to other willpower-dependent behaviours (e.g., substance abuse, smoking, video game addiction, and delinquent behaviour), engaging in exercise necessitates sustained self-control. Considering that greater self-control, associated in this study with a lower discounting tendency, is induced by nature exposure, exploring whether the effect of exposure to nature on reducing the discounting tendency could be generalised to other self-control behaviours is a question that merits future research.

In conclusion, our experiment, which involved actual physical activity, indicated that exposure to nature was linked with an increased propensity towards walking for health, and identified temporal discounting as a mechanism underlying this relationship. Participants exposed to images of urban landscapes demonstrated less walking activity than those exposed to images of natural landscapes, even when the health benefits of walking were explicitly communicated. In modern societies, where living and working environments are predominantly urban, our findings imply that the application of nature exposure (or immersion) may significantly promote engagement in exercise for health.

## Data Availability

The data that support the findings of this study are available in Mendeley at https://doi.org/10.17632/p67brh8r9v.1.

## Open scholarship

This article has earned the Center for Open Science badge for Open Data. The data are openly accessible at https://doi.org/10.17632/p67brh8r9v.1
